# Rational Design of an Epidermal Growth Factor Receptor Vaccine: Immunogenicity and Antitumor Research

**DOI:** 10.3390/biom14121620

**Published:** 2024-12-18

**Authors:** Yifei Liu, Zehui Liu, Zhongliang Zheng

**Affiliations:** College of Life Sciences, Wuhan University, Wuhan 430072, China; 2021202040159@whu.edu.cn (Y.L.); 2020202040159@whu.edu.cn (Z.L.)

**Keywords:** epidermal growth factor receptor, immunoinformatics, vaccine design, specific immune response

## Abstract

The epidermal growth factor receptor (EGFR) is frequently overexpressed in a variety of human epithelial tumors, and its aberrant activation plays a pivotal role in promoting tumor growth, invasion, and metastasis. The clinically approved passive EGFR-related therapies have numerous limitations. Seven EGFR-ECD epitope peptides (EG1-7) were selected through bioinformatics epitope prediction tools including NetMHCpan-4.1, NetMHCIIpan-3.2, and IEDB Consensus (v2.18 and v2.22) and fused to the translocation domain of diphtheria toxin (DTT). The A549 tumor model was successfully established in a murine mouse model. The vaccine was formulated by combining the adjuvants Alum and CpG and subsequently assessed for its immunogenicity and anti-tumor efficacy. DTT-EG (3;5;6;7) vaccines elicited specific humoral and cellular immune responses and effectively suppressed tumor growth in both prophylactic and therapeutic mouse tumor models. The selected epitopes EG3 (HGAVRFSNNPALCNV145-159), EG5 (KDSLSINATNIKHFK346-360), EG6 (VKEITGFLLIQAWPE398-412), and EG7 (LCYANTINWKKLFGT469-483) were incorporated into vaccines for active immunization, representing a promising strategy for the treatment of tumors with overexpressed epidermal growth factor receptor (EGFR). The vaccine design and fusion method employed in this study demonstrate a viable approach toward the development of cancer vaccines.

## 1. Introduction

The epidermal growth factor receptor (EGFR) is a transmembrane receptor protein that belongs to the type I tyrosine kinase family [1]. It is frequently overexpressed in human epithelial tumors, promoting tumor cell proliferation, inhibiting apoptosis, and facilitating angiogenesis [2,3]. EGFR has been associated with tumor progression, poor prognosis, and low survival rates [4], making it an attractive target for tumor therapy [5]. Currently approved clinical anti-EGFR drugs include tyrosine kinase inhibitors [6] as well as monoclonal antibodies such as cetuximab [7] and panitumumab [8]. However, limitations such as biocompatibility and pharmacokinetics of small molecule inhibitors [9], along with high costs, infusion reactions, and the emergence of drug resistance associated with monoclonal antibodies [10,11], restrict their widespread clinical application. CIMAvax-EGF consists of a chemical conjugate formed by epidermal growth factor (EGF) and the recombinant protein P64 from Neisseria meningitidis, along with an adjuvant (Montanide ISA51, VG) [12,13]. This vaccine induces the generation of antibodies against EGF, resulting in the depletion of circulating EGF and blocking the interaction between EGF and EGFR [14,15]. As an immunotherapy targeting epidermal growth factor (EGF), several clinical trials have been carried out in patients with advanced non-small-cell lung cancer (NSCLC), and the Cuban regulatory agency has approved CIMAvax-EGF as maintenance therapy after first-line treatment [15,16,17]. Despite the limited scope of EGFR-targeted immunotherapy to passive treatment approaches thus far employed successfully in clinics, it highlights the potential for active immunotherapy targeting EGFR. Vaccination-based strategies offer advantages including ease of administration and induction of durable tumor-specific immune responses. Recent advancements in cancer immunology have introduced treatment regimens that activate T cells and enhance immune responses effectively [18].

Epitope-based recombinant protein vaccines are composed of carrier proteins and tandemly arranged epitope peptide segments of tumor-associated antigens (TAAs). They are mainly taken up by antigen-presenting cells (APCs), processed, and presented on the cell surface via major histocompatibility complex (MHC) molecules [19,20]. CD8+ cytotoxic T cells recognize the peptides presented on MHC class I molecules, thereby being activated and expanded. These cytotoxic T lymphocytes (CTLs) possess the capability to selectively identify and target tumor cells that express tumor-associated antigens (TAAs), thereby infiltrating neoplasms and exerting their cytotoxic effects [20,21]. CD4+ helper T cells recognize the peptides presented on MHC class II molecules and provide assistance signals to other immune cells [22,23]. The peptides presented by dendritic cells (DCs) can activate B cells, resulting in the generation of antibodies against TAAs. These antibodies can directly bind to tumor cells, facilitate their destruction, and induce memory responses to enhance immune protection [24]. Therefore, the objective of certain epitope-based vaccines is to incorporate both MHC-I-restricted CTL epitopes and MHC-II-restricted CTL epitopes in order to achieve simultaneous stimulation of CTLs and Th cells [25,26]. Immunoinformatics has facilitated the development of several computer algorithm-based tools for predicting and analyzing epitopes [27,28,29]. When used independently in vaccine design, tumor-specific antigens or epitopes exhibit low immunogenicity and necessitate potent immune-stimulating adjuvants to effectively activate innate and acquired immunity [30,31]. The translocation domain of diphtheria toxin (DTT) can disrupt autoimmune tolerance through universal Th-cell epitopes [32,33]. EGFR exhibits high homology between humans and mice, and animals do not possess an endogenous immune response to EGFR prior to vaccination [34]. Furthermore, the rapid growth rate of tumors in mice suggests minimal endogenous immune responses toward tumor antigens.

T-cell epitopes interact with the grooves of MHC class I and class II molecules through R-group side chains [35]. This principle has been applied to various algorithms for expanding T-cell epitope localization [36,37]. At present, the prediction methods for MHC-binding peptides are mainly based on artificial neural networks (ANNs) [38], quantitative matrices (QM) [39], support vector machines (SVM) [40], etc., for predicting binding affinity. Due to the open ends of MHC class II binding grooves compared to the closed sides of MHC class I binding grooves, predicting MHC class II binding peptides poses greater challenges than predicting MHC class I binding peptides [41].

Alum and CpG are adjuvants that have been approved by the FDA for application in vaccines and clinical studies. Alum adjuvants have been extensively utilized in vaccines, such as those for diphtheria, tetanus, and hepatitis B. Alum adjuvant adsorbs antigens via a potent electrostatic interaction, maintaining a relatively high local concentration of antigens for a certain duration, enhancing the uptake and presentation of antigens by antigen-presenting cells (APCs) [42,43,44]. As efficient vaccine adjuvants, CpG oligodeoxynucleotides (ODNs) enhance specific humoral and cellular immune responses against antigens by activating Toll-like receptor 9 (TLR9) in dendritic cells and B cells and triggering an innate immune response characterized by the production of Th1 and pro-inflammatory cytokines [45,46,47]. CpG-B class, exemplified by CpG 1018, 1826, and 2007, represents the most prevalently utilized type in preclinical and clinical trials [48]. CpG 1826 is an extremely efficacious Th1 adjuvant with anti-tumor activity [49,50,51]. DTT-based antigens demonstrate immunogenicity when formulated with Alum adjuvant [49]. Alum + CpG adjuvant has the potential as a novel adjuvant. Compared with a single adjuvant, it can induce high antibody titers and significantly promote cellular immune responses and has the potential to be a combined adjuvant for human vaccines [44,52].

In this study, we chose NetMHCpan-4.1 [53] based on artificial neural networks (ANNs) and IEDB Consensus-2.18 [54] based on ANN and SMM to predict CTL epitopes. For the prediction of HTL epitopes, we employed NetMHCIIpan-3.2 [55], which utilizes ANNs, along with IEDB Consensus-2.22 [56], which incorporates ANNs and SMM methods. Subsequently, seven candidate epitopes for vaccine design named EG (1–7), encompassing both CTL and HTL epitopes with high homology to the mouse EGFR homologous sequence, were identified through screening processes. To construct the vaccine formulation, these seven recombinant proteins of DTT-EG (1-7) were developed using DTT as a carrier molecule in combination with adjuvants Alum and CpG. Furthermore, an A549 cell-based mouse tumor model was established to evaluate the immunogenicity as well as anti-tumor efficacy of the aforementioned DTT-EG vaccine through in vitro and in vivo experiments.

## 2. Materials and Methods

### 2.1. Ethical Statement

Animal studies were conducted in accordance with the guidelines set forth by the Ethics Committee of the Hubei Provincial Center for Disease Control and Prevention (ethics committee approval code and date: WHCDCIRB-K-2021063, 26 November 2021). Female Balb/c mice (6–8 weeks old) were procured from the Food and Drug Safety Evaluation Center at Hubei Center for Disease Control and Prevention. The animals were housed under controlled temperature and humidity conditions, with a 12 h light–dark cycle, and provided ad libitum access to food and water.

### 2.2. Cell Lines and Adjuvants

The human non-small-cell lung cancer cell line A549 cells were obtained from the Chinese Typical Culture Collection Center (CCTCC, Wuhan, China) and cultured in RPMI-1640 medium (Gibco, Grand Island, NY, USA) supplemented with 10% fetal bovine serum (FBS), 100 U/mL penicillin, and 100 μg/mL streptomycin (Gibco, USA). The cells were incubated at 37 °C under a humidified atmosphere of 5% CO_2_. The alumina adjuvant used was Imject^TM^ Alum (Thermo, Waltham, MA, USA). CpG ODN 1826 oligodeoxynucleotide sequence (TCCATGACGTTCCTGACGTT) was purchased from Suzhou Hongxun Biotechnology Co., Ltd. (Synbio Technologies, Suzhou, China). All chemical reagents used were of analytical grade.

### 2.3. Prediction of EGFR-ECD T-Cell Epitopes

T cell epitopes were predicted using NetMHCpan-4.1 [53] and IEDB Consensus-2.18 [54] for CD8+ T cells, while NetMHCIIpan-3.2 [55] and IEDB Consensus-2.22 [56] were used for predicting CD4+ T-cell epitopes. The prediction parameters of all epitope prediction servers are selected as the default values. The selection of HLA supertypes and alleles by each epitope prediction server during epitope prediction is presented in the following table (Table 1). PyMOL software 2.5.2 was employed to spatially localize the epitopes on EGFR-ECD. The predicted sequences were subjected to conservation and sequence homology analysis using NCBI BLAST (https://blast.ncbi.nlm.nih.gov/smartblast/?LINK_LOC=BlastHomeLink (accessed on 17 September 2021)). Subsequently, the population coverage of the predicted epitope peptide segments was conducted using IEDB Population Coverage [57]. The prediction options of MHC class I and II combinations were chosen, and the predicted population was set as World.

Subsequently, IEDB NetMHCIIpan 4.1 BA [58] and NetMHCpan-4.1 [53] were employed to determine whether these epitopes obtained after prediction and screening were recognized by mouse MHC alleles. The prediction parameters of all epitope prediction servers are all selected as the default values. IEDB NetMHCIIpan 4.1 BA predicted whether these epitopes could bind to BALB/c mouse MHC-II alleles (H-2-IAd and H-2-IEd), and NetMHCpan-4.1 predicted whether there were 9-amino acid-long sequences among these epitopes that were recognized by BALB/c mouse MHC-I alleles (H-2-Dd, H-2-Kd, and H-2-Ld).

### 2.4. DTT-EG Design and Expression Vector Construction

Seven 15-amino acid peptide fragments, named EG1-EG7, were constructed at the C-terminal end of DTT through a single tandem repeat using the GS linker. The resulting recombinant proteins were designated as DTT-EG1 to DTT-EG7. The DTT sequence corresponds to amino acid residues 202-373 of the diphtheria toxin protein [33]. The GS linker (GGTGGTGGTGGTAGTGGTGGTGGTGGTAGT) is a commonly used flexible linker that maintains the independent structure and function of both carrier protein and fused peptide or protein without causing immune reactivity [59]. Wuhan Gene Create Biological Engineering Co., Ltd. (GENECREATE, Wuhan, China) chemically synthesized seven recombinant DNA fragments, which were then cloned into pET-28(a)+ vector.

### 2.5. DTT-EG Expression and Purification

The seven expression vectors pET-28(a)+-DTT-EG (1, 2, 3, 4, 5, 6, 7) were transformed into *E. coli* BL21(DE3), and a single colony was inoculated into 100 mL of LB medium containing kanamycin resistance at a concentration of 50 μg/mL. The culture was incubated overnight at 37 °C and shaken at a speed of 200 rpm. Subsequently, the culture was transferred to a larger volume of LB medium (1L) supplemented with kanamycin resistance at a concentration of 100 μg/mL and cultured until the optical density at wavelength λ = 600 nm (OD600) reached approximately 0.8. Then IPTG (isopropyl-beta-D-thiogalactopyranoside) was added to achieve a final concentration of 100 μmol/L, and the culture was further incubated for an additional duration of 20 h under conditions maintained at a temperature of 16 °C while shaking continuously at a speed of 200 rpm. The cells were harvested by centrifugation and subsequently resuspended in Tris buffer (25 mM Tris-HCl, 100 mM NaCl, 10% glycerol, pH 7.5). Subsequently, the cells were disrupted using ultrasonication in an ice water bath, followed by centrifugation at 4 °C and 10,000 rpm for 30 min. The supernatant after centrifugation was filtrated through a 0.45 μm membrane and then successively purified by PC004-azybon′Ni-HisTalon μSphere (Sinopae, Wuxi, China) and PC024 HiTalon Q-μSphere (Sinopae, Wuxi, China). The supernatant protein was loaded onto a Ni-affinity chromatography column and then subjected to gradient elution using imidazole. The target protein was verified through 12% SDS-PAGE analysis. Subsequently, the elution solution containing the target protein was diluted, and the protein was loaded onto a Q column (anion-exchange column) for NaCl gradient elution. Purity was determined by 12% SDS-PAGE, concentrated by centrifugation, and quantified by the BCA method.

### 2.6. Mouse Immunity

The mice were immunized using a vaccination protocol consisting of three consecutive injections [49,60,61]. The experimental group was immunized with 50 μg of recombinant protein DTT-EG, 300 μg of Alum, and 30 μg of CpG, which were added into a 200 μL PBS buffer (Gibco, USA), respectively. The control group received immunization with either PBS or a mixture of 50 μg DTT, 300 μg Alum, and 30 μg CpG. The immunization regimen involved subcutaneously injecting a volume of 200 μL vaccine into the axilla region of female Balb/c mice (6–8 weeks old) on days 1, 12, and 24. One week after the third immunization, blood samples were collected from the orbital area of mice. The samples were centrifuged to obtain the upper serum layer, which was aliquoted and stored at −80 °C. Similarly, one week following the third immunization, the spleen was excised from the mice and splenic lymphocytes were isolated. The isolated mouse spleen lymphocytes were immediately utilized for the subsequent cell response experiments on the same day.

### 2.7. ELISA Antibody Detection

The serum of immunized mice was subjected to an enzyme-linked immunosorbent assay (ELISA) for the detection of antibodies against DTT and EGFR. A 96-well plate was coated with 100 ng of DTT or Recombinant Human EGFR Protein (ABclonal, Wuhan, China) in 100 μL of coated buffer (0.05 M carbonate buffer, pH 9.6), followed by overnight incubation at 4 °C. To block non-specific binding sites, each well was treated with a blocking solution prepared by adding 200 μL of 1% BSA PBS-T (containing 0.05% Tween20) and incubating at 37 °C for 1 h. The immune mouse serum was diluted 100 times at a ratio of 1:100 with PBS-T containing 1% BSA. Subsequently, 100 µL of the diluted serum was dispensed into the designated wells of the ELISA microplate and incubated at 37 °C for a duration of 1 h. HRP-conjugated Affinipure Goat Anti-Mouse IgG (H+L) antibody (Proteintech, Wuhan, China), diluted to a ratio of 1:10,000 in the same buffer as above, was then added to each well at a volume of 100 μL and further incubated at 37 °C for an additional hour. Afterward, TMB substrate display solution was introduced into each well without light exposure and allowed to react for 15 min at room temperature. The reaction was stopped by adding 50 μL of 2M H_2_SO_4_ to each well. Finally, the absorbance at 450 nm was measured using a Cytation3 multifunction plate reader with the corrected wavelength set as 630 nm. The antibody titer was defined as the highest dilution that resulted in an optical density (OD) value greater than 0.2.

### 2.8. T-Cell Proliferation Detection

Female Balb/c mice (*n* = 4) were immunized following the protocol described in Section 2.6. titled “Mouse Immunity”. The spleens of female Balb/c mice were harvested one week after the third immunization and mechanically dissociated through a 70-mesh cell sieve. The cell proliferation experiment was conducted on the same day as the isolation of spleen lymphocytes. A single-cell suspension was prepared using PBS, followed by treatment with erythrocyte lysate (Solarbio, Beijing, China) to remove erythrocytes. Lymphocytes were then resuspended in RPMI-1640 medium supplemented with 10% FBS and 10 ng/mL rhIL-2 (ABclonal, Wuhan, China). Subsequently, 100 μL of the lymphocyte suspension at a density of 1 × 10^5^ cells/well was seeded into each well of a 96-well plate. Spleen lymphocytes from mice immunized with PBS and DTT vaccine served as negative controls. In the experimental group, wells were stimulated with DTT-EG protein at a concentration of 30 μg/mL, while in the control group, wells were stimulated with either DTT or PBS at the same concentration. Corresponding unstimulated cells served as additional controls. After 72 h of cell stimulation culture, in accordance with the instructions of the Cell Counting Kit-8 (biosharp, Anhui, China), 20 µL of the CCK-8 detection solution was added to each cell culture well. The cells were further incubated in the cell culture incubator for 30 min. The absorbance value at 450 nm of each well was measured using an enzyme-labeled instrument every 30 min, and the time points with a relatively appropriate absorbance range were selected for recording. Absorbance at 450 nm was measured using a Cytation3 multifunction enzyme-linked immunosorbent assay apparatus with a correction wavelength set at 650 nm. The stimulation index (SI) is determined by the ratio of the optical density (OD) of the cell culture wells stimulated by the protein at 450 nm to that of the wells not stimulated by the protein, namely, SI = [(OD (stimulated by protein) − OD (blank))/(OD (unstimulated) − OD (blank))] × 100%. Under the stipulated conditions, the more and faster the cells proliferate upon stimulation, the greater the OD450 value detected after CCK-8 staining, and the larger the SI value will be.

### 2.9. Cytotoxicity Testing

The immunization protocol for mice, the time point of spleen lymphocyte extraction from immunized mice, and the method of separation and extraction were conducted in accordance with Section 2.8 for T-cell proliferation detection. The isolated splenic lymphocytes were resuspended in a medium containing 10% FBS, RPMI-1640, and 10 ng/mL rhIL-2. Subsequently, they were stimulated with either 30 μg/mL DTT-EG stimulating protein, 30 μg/mL DTT alone, or PBS for a duration of 72 h. The spleen lymphocytes, which were stimulated and cultured for 72 h, were utilized as effector cells. To assess cytotoxicity, effector spleen lymphocytes stimulated by DTT-EG protein were co-cultured with A549 target cells (3000 cells per well) in 96-well plates at various effector-to-target cell ratios (50:1, 20:1, and 10:1) for a duration of 8 h. The co-culture was conducted in wells containing a total cell culture medium volume of 200 μL each, maintained at a temperature of 37 °C with a CO_2_ concentration of 5%. Following this incubation period, the supernatant from each well was collected (120 μL) to assess cytotoxicity using the LDH Cytotoxicity Assay Kit (Beeyotime, Wuhan, China). The killing rate observed in splenic lymphocytes immunized with PBS and DTT vaccine served as the negative control.

### 2.10. Interferon-γ Detection

According to the aforementioned experimental approach (Section 2.8 T-cell proliferation detection), one week after the third immunization, splenic lymphocytes from mice were collected at a volume of 500 μL per well and inoculated into 24-well plates with a cell density of 5 × 10^5^ cells/well. The experimental groups were stimulated with the corresponding recombinant protein DTT-EG at a concentration of 30 μg/mL, while the control group was treated with PBS or DTT at a concentration of 30 μg/mL. Subsequently, the cells were cultured under conditions of 37 °C and 5% CO_2_. At the designated time point, the supernatant was harvested for analysis, and a Mouse IFN-gamma ELISA Kit (ABclonal, Wuhan, China) was employed to measure the concentration of IFN-γ.

### 2.11. Immunohistochemical Analysis of Spleen in Immunized Mouse

Female Balb/c mice (*n* = 4) were immunized according to the protocol described in Section 2.6. titled “Mouse Immunity”, with their spleens taken one week after the third immunization. The spleens were fixed in a neutral tissue fixation solution containing 4% paraformaldehyde and subsequently sent to Wuhan Servare Biotechnology Co., Ltd. (Servicebio, Wuhan, China) for paraffin embedding and preparation of tissue sections. These sections were then stained with an anti-mouse CD4- and CD8-specific antibody and quantitatively analyzed using ImageJ software 1.54g, with the selection of five different representative fields at a magnification of 10× for each section.

### 2.12. Mouse Tumor Model Experiment

**Figure 1:**
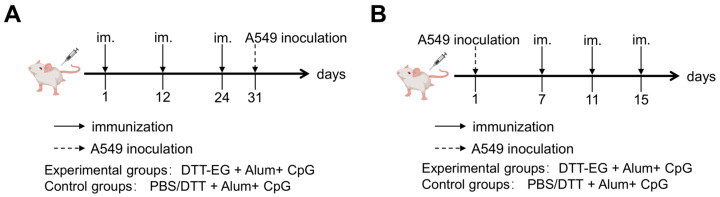
Immunization with DTT-EG vaccines in combination with tumor cell injection protocol. (**A**) Prophylactic tumor model: tumor cell injection and vaccine immunization protocol (mouse diagram by medpeer.cn) and (**B**) therapeutic tumor model: tumor cell injection and vaccine immunization protocol (mouse diagram by medpeer.cn (accessed on 9 June 2024)).

For the prophylactic tumor model (Figure 1A), female Balb/c mice (6–8 weeks old, *n* = 6) were subcutaneously immunized with DTT-EG+ Alum+ CpG vaccine on days 1, 12, and 24. Control groups receiving PBS+ Alum+ CpG and DTT+ Alum+ CpG were also established. One week after the third immunization, a total of 2.5 × 10^7^ A549 cells resuspended in 150 μL PBS were subcutaneously injected into the right axilla of each mouse. Daily monitoring and recording of the tumor growth are conducted. The longest diameter (L) and the shortest transverse diameter (W) of the tumor are measured using a vernier caliper. The tumor volume is calculated by multiplying the longest diameter by the square of the shortest transverse diameter and dividing the result by 2, that is, the tumor volume = [(L) × (W)^2^]/2. On day 14 or 16 after tumor cell inoculation, mice were sacrificed for the collection of tumors to measure their weight and perform immunohistochemical analysis.

For the therapeutic tumor model (Figure 1B), 2.5 × 10^7^ A549 cells were subcutaneously injected into the right axilla of female Balb/c mice (6–8 weeks old, *n* = 6) in a volume of 150 μL PBS. Subsequently, each immune vaccine was administered on the 7th day post-cell injection, followed by two additional immunizations at 4-day intervals. Tumor size was monitored and measured as previously described. The tumor volume is calculated by multiplying the longest diameter by the square of the shortest transverse diameter and dividing the result by 2, that is, the tumor volume = [(L) × (W)^2^]/2. On the 23rd day after tumor cell inoculation, mice were euthanized and tumors were collected for weight measurement and immunohistochemical analysis.

### 2.13. Immunohistochemical Analysis of Tumor Tissues

The tumor tissues were fixed in a neutral tissue fixation solution containing 4% paraformaldehyde and subsequently sent to Wuhan Servare Biotechnology Co., Ltd. (Servicebio, Wuhan, China) for paraffin embedding and preparation of tissue sections. The sections were stained with Recombinant Anti-CD4 antibody (Servicebio, Wuhan, China) and Anti-CD8 alpha Mouse mAb (Servicebio, Wuhan, China), respectively, and quantitative analysis was conducted using ImageJ software 1.54g. Five different representative fields of view were selected for each section at a magnification of 20 times. The tumor tissue sections were subjected to H&E staining, and representative regions were chosen from each section under a magnification of 20×.

### 2.14. Statistical Analysis

The statistical significance analysis was conducted using GraphPad Prism9 software. Data were presented as mean ± SD (standard deviation), and multiple comparisons were performed using one-way ANOVA or two-way ANOVA. Significance levels were denoted as * *p* < 0.05, ** *p* < 0.01, *** *p* < 0.001, **** *p* < 0.0001, while “ns” indicated no significant difference.

## 3. Results

### 3.1. Design of DTT-EG Vaccine

**Figure 2:**
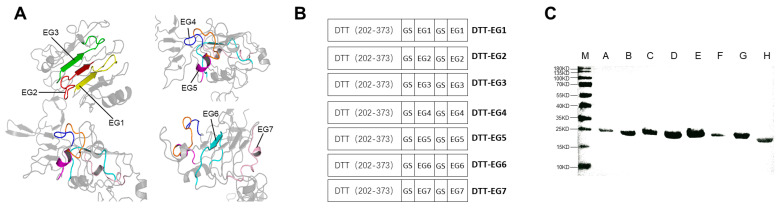
The predicted epitope EG in EGFR-ECD is demonstrated, along with the design and expression purification of DTT-EG utilizing DTT as a vector. (**A**) Displaying the EG epitope within EGFR-ECD (EGFR PDB id:3njp): EG1 is represented by a yellow sequence, EG2 by a red sequence, EG3 by a green sequence, EG4 by a blue and orange sequence, EG5 by an orange and purple sequence, EG6 by a cyan sequence, and EG7 by a pink sequence. (**B**) Design of DTT-EG tandem recombinant protein. DTT (202–373) denotes the amino acid fragment spanning from 202 to 373 of the DTT protein. The epitope prediction tool identified seven human-specific epitope peptides in the form of EG1, EG2, EG3, EG4, and E5G5G6G7 consisting of 15 amino acid residues each. GS represents the GS-linker sequence (GGTGGTGGTGGTAGTGGTGGTGGTGGTAGT). (**C**) Analysis of purified recombinant protein using 12% SDS-PAGE (M: Marker; A: DTT-EG1; B: DTT-EG2; C: DTT-EG3; D: DTT-EG4; E: DTT-EG5; F: DTT-EG6; G: DTT-EG7; H: DTT).

In order to predict HTL epitopes containing CTL epitopes in EGFR-ECD for the construction of an epitope-based vaccine that can simultaneously stimulate Th cells and CTL, we employed indirect methods for T-cell epitope prediction. Specifically, NetMHCpan-4.1 [53] and IEDB Consensus-2.18 [54] were selected to predict the CTL epitopes (MHC class I binding peptides), while NetMHCIIpan-3.2 [55] and IEDB Consensus-2.22 [56] were chosen to predict the HTL epitopes (MHC class II binding peptides). The length of MHC class I binding peptides ranged from 8 to 11 amino acids, with a preference for 9-amino acid-long peptides by most MHC class I molecules [62]. The prediction parameters for NetMHCpan-4.1 were set as IC50 < 50 nm and %Rank < 0.5%, while default options were used for IEDB Consensus-2.18. The combined results from both tools identified eight commonly predicted CTL epitope peptides with a length of 9 amino acids: YVLIALNTV88-96, MYYENSYAL111-119, YENSYALAV113-121, AVRFSNNPA147-155, GEFKDSLSI343-351, NATNIKHFK352-360, KEITGFLLI399-407, and YANTINWKK471-479 (Table 2). For MHC class II binding peptides in EGFR-ECD, their lengths mostly ranged from 14 to 18 amino acids with a representative length of 15 amino acids [63]. The prediction parameters for NetMHCIIpan-3.2 were set as IC50 < 500 nm, while the prediction criteria for IEDB Consensus-2.22 were defined as %Rank < 10%. By combining the results from both methods, a total of 7 HTL epitope peptides with a length of 15 amino acids, containing the identified CTL epitopes, were selected. These peptides were named EG1 (YVLIALNTVERIPLE88-102), EG2 (RGNMYYENSYALAVL108-122), EG3 (HGAVRFSNNPALCNV145-159), EG4 (IGIGEFKDSLSINAT340-354), EG5 (KDSLSINATNIKHFK346-360), EG6 (VKEITGFLLIQAWPE398-412), and EG7 (LCYANTINWKKLFGT469-483) based on their respective positions in the sequence alignment analysis (Table 3). Notably, all 7 HTL epitopes containing CTL epitopes were found within the domain of EGFR-ECD (EGFR PDB id: 3njp) (Figure 2A). To assess the homology between these seven peptides and corresponding ones in EGFR-Mouse, NCBI BLAST was utilized revealing homologies of EG1-100.00%, EG2-73.33%, EG3-85.71%, EG4-93.33%, EG5-93.33%, EG6-93.33%, and EG7-100.00%, respectively (Table 3). Meanwhile, the results of NCBI BLAST indicated that the seven peptide segments, namely, EG1-7, are highly conserved sequence fragments of EGFR, and there is no cross-reactivity with other human proteins. The population coverage of 7 epitope peptides worldwide was predicted using IEDB Population Coverage [57] (Table 3). Since HLA-DRB5-01:01 was not included in the database, the peptide segment EG7 only contained the prediction results for HLA-A-68:01 with the YANTINWKK epitope. These seven identified peptides encompass HLA-I-restricted CTL epitopes recognized by CD8+ T cells as well as HLA-II-restricted CTL epitopes recognized by CD4+ T cells.

In order to clarify whether the epitopes EG1-7 obtained after prediction and screening could be recognized by mouse MHC alleles, predictions were conducted using IEDB NetMHCIIpan 4.1 BA [58] and NetMHCpan-4.1 [53], respectively (Table 4). The prediction results of NetMHCpan-4.1 were presented in accordance with its default output, while the prediction results of IEDB NetMHCIIpan 4.1 BA were based on IC50 values (IC50 < 50 nM-high affinity, <500 nM-intermediate affinity, and <5000 nM-low affinity). The prediction results of recognition of epitopes EG1-7 by MHC haplotypes of Balb/c mice showed that, for MHC-II class H-2-IAd and H-2-IEd, except that epitope EG4 could only be recognized by H-2-IAd, the remaining six epitopes could be recognized by both H-2-IAd and H-2-IEd. The prediction results of binding of epitopes EG1-7 to MHC-II class haplotypes indicated that all bindings were weak. The recognition prediction results for MHC-I class H-2-Dd, H-2-Kd, and H-2-Ld showed that, except that no CTL epitope was predicted in epitope EG4, CTL epitopes with a length of 9 amino acids that could be recognized by the corresponding MHC-I class haplotypes were predicted in the remaining six epitopes.

Prior to vaccination, no endogenous immune response against EGFR existed in the animal body [34]. To overcome immune auto-tolerance, we constructed DTT-EG1 to DTT-EG7 by fusing the selected peptide segment EG to the C-terminal of DTT via a flexible linker peptide GS linker [64] (Figure 2B). The *E. coli* expression vector pET-28a (+)-DTT-EG was then created and used for expressing seven His-labeled recombinant proteins in soluble form, which were purified using a Ni-NTA column and anion exchange column before being analyzed by 12% SDS-PAGE after purification (Figure 2C).

### 3.2. Detection of DTT-EG-Induced Specific Antibody Response in Mouse

**Figure 3:**
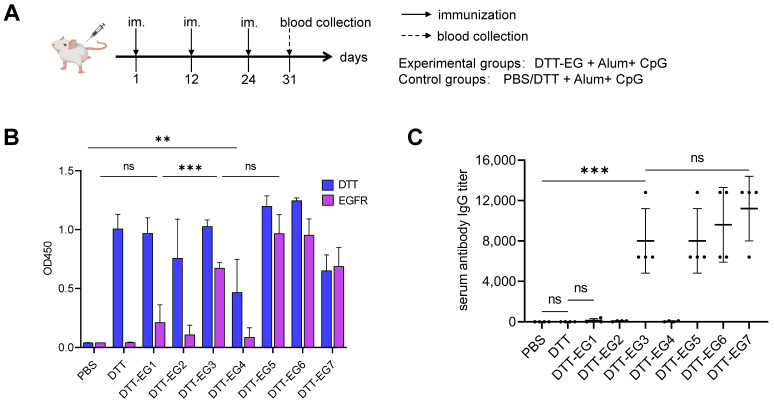
Serum antibodies were detected using enzyme-linked immunosorbent assay (ELISA) following immunization of mice with the DTT-EG tandem recombinant protein. (Significance levels were denoted as ** *p* < 0.01, *** *p* < 0.001, while “ns” indicated no significant difference). (**A**) Schematic representation of the immunization protocol for mice (mouse diagram by medpeer.cn (accessed on 9 June 2024)). (**B**) ELISA analysis was conducted on the serum of immunized mice, with the coated proteins being DTT or EGFR. (**C**) The titer of antibodies against EGFR in the mouse serum following vaccination was determined by ELISA.

The mice were immunized following the established protocol in a manner consistent with standard scientific practice (Figure 3A). One week after the third immunization, serum antibody responses of immunized mice to DTT and EGFR were assessed by ELISA (Figure 3B, Supplementary Table S1). The ELISA results demonstrated that specific antibodies targeting DTT were detectable in the sera of immunized mice in all groups except the PBS control group, suggesting that the recombinant proteins DTT-EG possess normal functional DTT domains. In the control group, the PBS and DTT groups showed no detectable endogenous anti-EGFR antibodies present in mouse serum samples. However, a strong anti-EGFR antibody response was observed among experimental groups including DDT-EG3, DDT-EG5, DDT-EG6, and DDT-EG7 with no significant differences between these four groups. The antibody titers of the experimental groups exhibiting robust anti-EGFR antibody responses were 8000 ± 3200 in the DTT-EG3 group, 8000 ± 3200 in the DTT-EG5 group, 9600 ± 3695 in the DTT-EG6 group, and 11,200 ± 3200 in the DTT-EG7 group, respectively. There was no significant difference observed among these four groups (Figure 3C, Supplementary Table S1). These results demonstrated that while epitopes within the DTT-EG1, DTT-EG2, and DTT-EG4 groups failed to elicit specific humoral immunity against EGFR, those within the DTT-EG3, DTT-EG5, DTT-EG6, and DTT-EG7 groups successfully induced specific humoral immune responses against EGFR.

### 3.3. Analysis of Cell Responses Induced by DTT-EG

**Figure 4:**
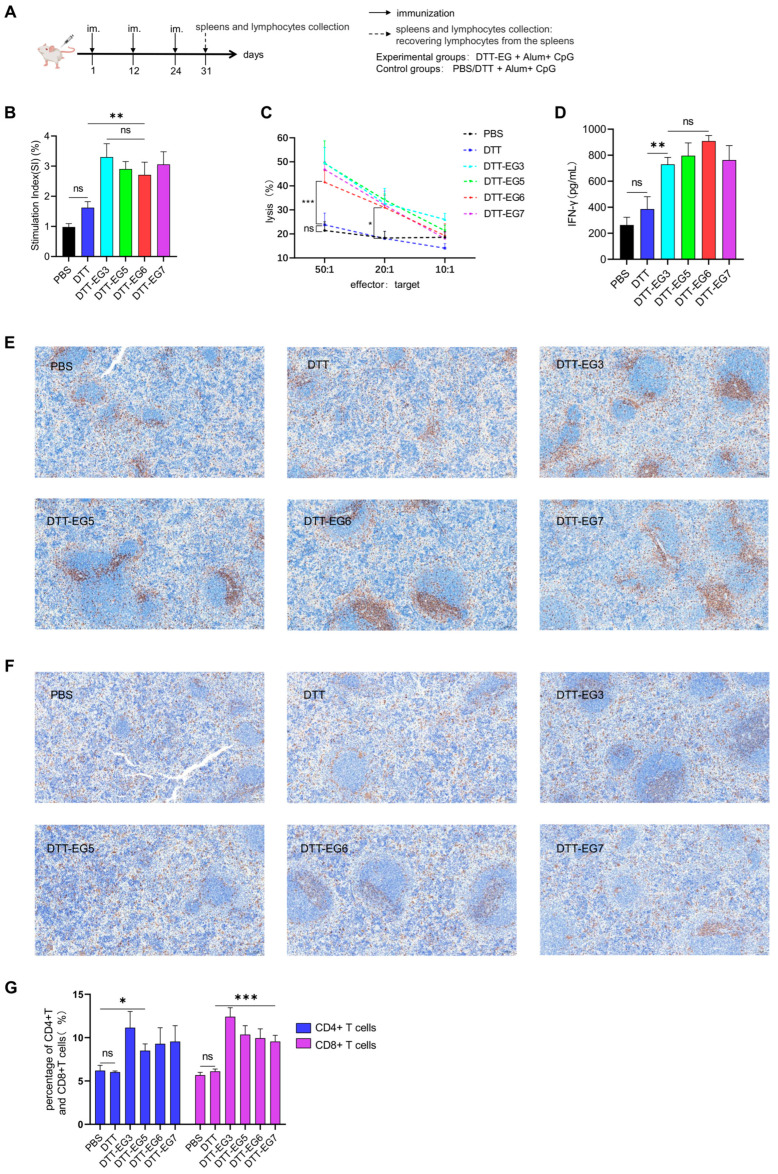
The splenic lymphocytes of immunized mice were tested for cell proliferation, toxicity, and interferon-gamma release, and the spleens were tested for CD4 and CD8 immunohistochemistry. (Significance levels were denoted as * *p* < 0.05, ** *p* < 0.01, *** *p* < 0.001, while “ns” indicated no significant difference). (**A**) Schematic representation of the immunization protocol for mice (mouse diagram by medpeer.cn (accessed on 9 June 2024)). (**B**) Cell proliferation detection experiment by CCK-8 method. (**C**) Cell cytotoxicity detection experiment by lactate dehydrogenase method. (**D**) Detection of IFN-γ release by ELISA method. (**E**) The spleen of immunized mice was stained by immunohistochemistry with anti-CD4 specific antibody. (**F**) The spleen of immunized mice was stained by immunohistochemistry with anti-CD8 specific antibody. (**G**) CD4+ T and CD8+ T-cell density was quantified using ImageJ.

To investigate the cellular memory response following immunization with the DTT-EG vaccine, splenic lymphocytes were isolated from immunized mice and subsequently stimulated in vitro with corresponding DTT-EG proteins. The spleens and splenic lymphocytes of mice were isolated one week after the third immunization in accordance with the immunization schedule (Figure 4A). The subsequent cell experiments were conducted immediately following the isolation and extraction of splenic lymphocytes. In cellular experiments, the individual tandem epitope EG should be employed. We have constructed the individual tandem EG on expression vectors before. However, after numerous attempts of various expressions, we failed to obtain the individual tandem EG protein successfully. Thus, we selected DTT + Alum + CpG vaccine as one of the immunization control groups and DTT-EG + Alum + CpG as the experimental group. The cellular responses triggered by the epitope EG were investigated through the indirect experimental approach of in vitro stimulation with DTT or DTT-EG protein.

The cell proliferation response was assessed using the CCK-8 assay. The stimulation indices (SI) of each group demonstrated that, compared with the PBS and DTT control groups, the spleen lymphocytes of immunized mice in the DTT-EG3, DTT-EG5, DTT-EG6, and DTT-EG7 groups proliferated significantly after stimulation, and there was no obvious difference among the four experimental groups (Figure 4B, Supplementary Table S2). The average stimulation index of these four groups was approximately three times higher than that of the PBS control and nearly two times higher than that of the DTT treatment. The results demonstrated that the epitopes EG3, EG5, EG6, and EG7 specifically elicited proliferation of spleen lymphocytes in mice immunized with their respective vaccines. This suggests that DTT-EG3, DTT-EG5, DTT-EG6, and DTT-EG7 vaccines induced specific T-cell memory responses against the corresponding epitopes.

In order to investigate the cytotoxic T-lymphocyte response following immunization with the DTT-EG vaccine, splenic lymphocytes from mice immunized with the DTT-EG vaccine were stimulated in vitro with the corresponding DTT-EG protein. The activated lymphocytes were then co-cultured with A549 tumor cells, and the cytotoxicity of the treated lymphocytes was assessed by measuring LDH release. The results of the cell lysis rates of stimulated lymphocytes as effector cells and A549 cells as target cells at the effector: Target ratios of 50:1 and 20:1 demonstrated that the cytotoxicity of spleen lymphocytes from DTT-EG3, DDT-EG5, DDT-EG6, and DDT-EG7 immunized mice was enhanced compared with the PBS and DDT control groups (Figure 4C, Supplementary Table S2). The findings demonstrated that mice immunized with DTT-EG3, DTT-EG5, DTT-EG6, and DTT-EG7 vaccines exhibited enhanced cytotoxicity in spleen lymphocytes and elicited robust cytotoxic immune responses against A549 cells.

To investigate the induction of IFN-γ release following immunization with the DTT-EG vaccine, splenic lymphocytes from mice immunized with the DTT-EG vaccine were stimulated in vitro with the corresponding DTT-EG protein. After 72 h stimulation culture, the culture supernatants were collected, and the contents of INF-γ in each group were analyzed using the INF-γ ELISA kit (Figure 4D, Supplementary Table S2). No significant differences were observed among these experimental groups with respect to IFN-γ release levels. The results demonstrated that, in comparison to the control group, spleen lymphocytes from mice immunized with DTT-EG3, DTT-EG5, DTT-EG6, and DTT-EG7 vaccines exhibited an increased secretion of INF-γ upon in vitro stimulation with corresponding DTT-EG proteins.

In order to investigate the cell density of CD4+ T and CD8+ T lymphocytes in the spleen of mice immunized with the DTT-EG vaccine, immunohistochemical analysis was conducted on spleen tissues (Figure 4E,F). The results of immunohistochemical staining for CD4 and CD8 demonstrated that the proportion of CD4+ T cells and CD8+ T cells in the spleen of immunized mice was significantly increased in the DTT-EG3, DTT-EG5, DTT-EG6, and DTT-EG7 groups compared to the control group PBS group and DTT group, with a particularly notable increase in CD8+ T cells (Figure 4G, Supplementary Table S2). These findings suggest that epitopes EG3, EG5, EG6, and EG7 in DTT-EG3, DTT-EG5, and DTT-EG7 are capable of eliciting specific proliferation of CD4+ T and CD8+ T lymphocytes in the spleen of immunized mice.

### 3.4. Effects of DTT-EG Vaccine in Mouse Tumor Models

**Figure 5:**
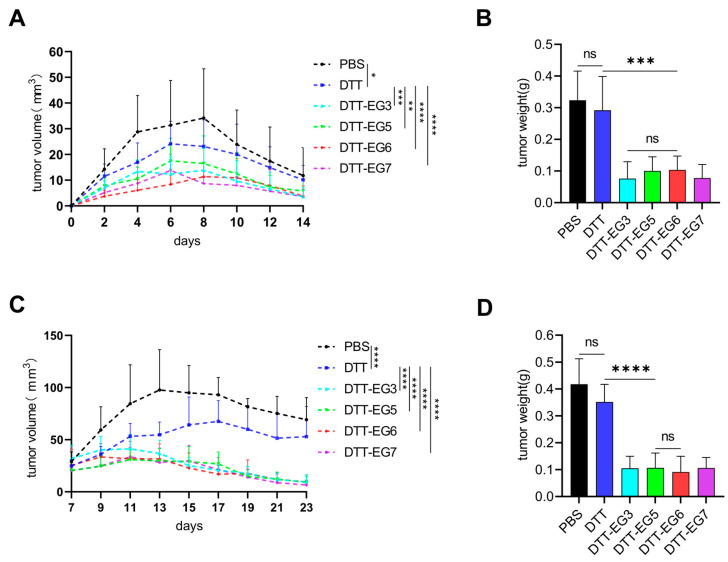
Antitumor effects of the DTT-EG vaccine in a prophylactic and therapeutic mouse A549 tumor model. (Significance levels were denoted as * *p* < 0.05, ** *p* < 0.01, *** *p* < 0.001, **** *p* < 0.0001, while “ns” indicated no significant difference). (**A**) prophylactic tumor model tumor growth curve, (**B**) prophylactic tumor model tumor weight, (**C**) therapeutic tumor model tumor growth curve, and (**D**) therapeutic tumor model tumor weight.

To investigate the inhibitory effect of DTT-EG vaccine-induced immunization on tumor growth, a total of 2.5 × 10^7^ A549 cells were injected into the right axilla of mice on the 7th day following the third immunization. The tumor growth curve was recorded, and tumors were weighed on the 14th day post-tumor cell injection. After analyzing the tumor growth curve, it was observed that tumor growth was significantly inhibited in all four experimental groups as compared to the control group (Figure 5A). There were no significant differences observed among these experimental groups regarding tumor weight measurements. Compared to both PBS and DTT groups, a decrease was observed in tumor weights for the DTT-EG3, DTT-EG5, DTT-EG6, and DTT-EG7 groups (Figure 5B, Supplementary Table S3); this observation was consistent with volume measurements obtained from growth curve analysis. The results demonstrated that prophylactic administration of DTT-EG3, DTT-EG5, DTT-EG6, and DTT-EG7 vaccines exerted inhibitory effects on tumor growth in the mouse model.

The tumor model was established in Balb/c mice using the A549 human cell line. In the pre-experiment of the therapeutic model, immunizations were carried out at the predetermined times on the 1st, 12th, and 24th days after tumorigenesis, respectively. However, due to significant regression of the tumor prior to completing three consecutive immunizations, subsequent treatment model experiments have modified the vaccination interval considering both the innate immune response of mice and tumor characteristics. To investigate the therapeutic efficacy of DTT-EG vaccine-induced immunization against tumors, immunization was conducted on the 7th day following the injection of 2.5 × 10^7^ A549 cells into the right axilla of mice. The tumor growth curve was recorded, and tumor weight was measured on the 16th day post-tumor cell injection. Compared to the PBS and DTT groups, the tumor volumes in the DTT-EG3, DTT-EG5, DTT-EG6, and DTT-EG7 groups were significantly reduced (Figure 5C). The reduction in tumor weights observed in the DDT-EG3, DDT-EG5, DDT-EG6, and DDT-EG7 groups was consistent with our findings from volume measurements on growth curves when compared to both PBS and DTT control groups, with no significant differences observed among these four experimental groups in terms of tumor weights (Figure 5D, Supplementary Table S3). The findings suggest that candidate immune vaccines, namely, DTT-EG3, DTT-EG5, DTT-EG6, and DTT-EG7, possess the ability to elicit therapeutic antitumor immunity in a murine model of therapy-induced tumors.

The results obtained from the prophylactic and therapeutic mouse tumor models demonstrate that DTT-EG3, DTT-EG5, DTT-EG6, and DTT-EG7 vaccines exhibit significant inhibitory effects on tumor growth.

### 3.5. Effects of DTT-EG Vaccine on Infiltration of CD8+ T Cells into the Tumor Microenvironment

**Figure 6:**
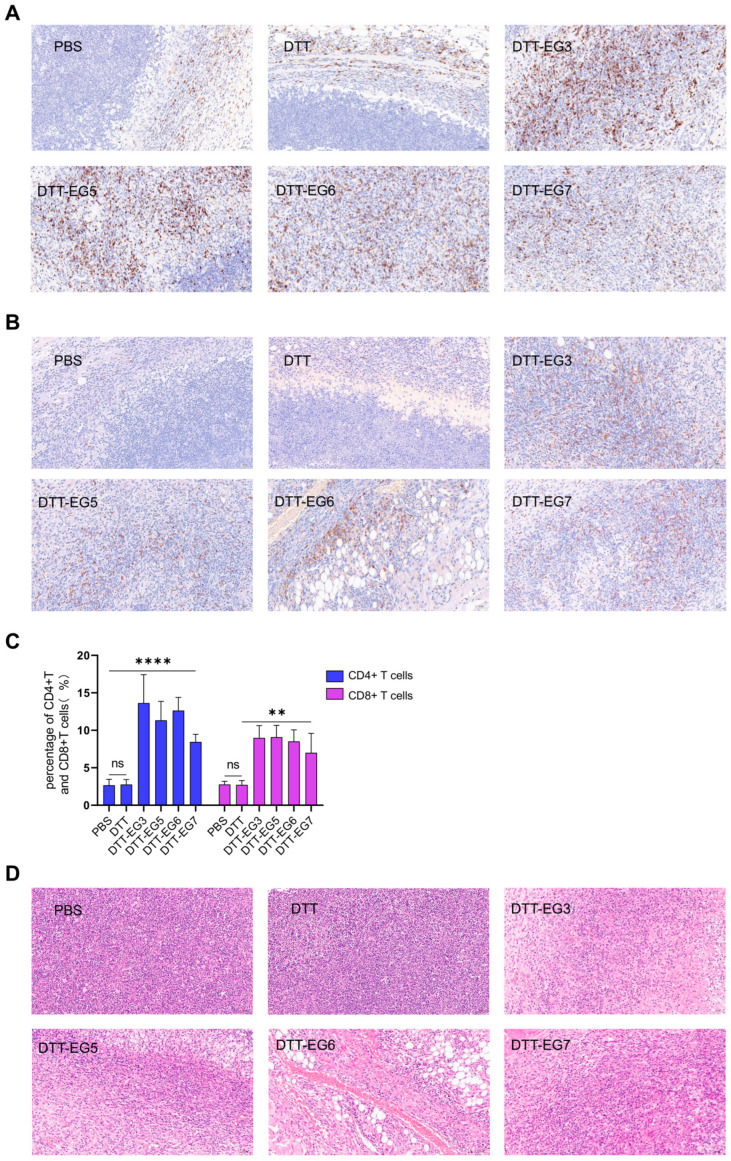
The DTT-EG vaccine modulates the infiltration of CD8+ T cells and induces necrosis within the intratumoral tissue. (Significance levels were denoted as ** *p* < 0.01, **** *p* < 0.0001, while “ns” indicated no significant difference). (**A**) Tumor tissues underwent immunohistochemical staining using an anti-CD4 specific antibody. (**B**) Tumor tissues underwent immunohistochemical staining using an anti-CD8 specific antibody. (**C**) CD4+ T and CD8+ T cell density was quantified using ImageJ. (**D**) Revealing the histopathological features of tumor tissue through hematoxylin and eosin (H&E) staining.

Tumor-infiltrating lymphocytes serve as prognostic indicators for numerous cancer therapies. The tumor tissues were subjected to immunohistochemical staining for CD4 and CD8 (Figure 6A,B). The results of anti-CD4 and CD8 staining of tumor tissues demonstrated that compared with the PBS and DTT control group, inoculation of DTT-EG3, DTT-EG5, DTT-EG6, and DTT-EG7 vaccines led to a significant increase in the proportion of CD4+ T cells in tumor tissues (Figure 6C, Supplementary Table S4). Additionally, there was a notable enhancement in the infiltration of CD8+ T cells in tumor tissues (Figure 6C, Supplementary Table S4). The occurrence of necrosis in tumor tissues was infrequent among mice immunized with PBS and DTT, whereas evident tumor necrosis was observed in the tumor sections of mice immunized with DTT-EG3, DTT-EG5, DTT-EG6, and DTT-EG7 vaccines when compared to the PBS and DTT groups (Figure 6D). These findings indicate that immunization with DTT-EG vaccines in the four experimental groups elicited activation of specific CD8+ T cells in mice, which subsequently infiltrated tumor tissues as effector CD8+ T cells and contributed to tumor lysis.

## 4. Discussion

Currently, EGFR has emerged as a prominent target for cancer therapy due to its overexpression or abnormal expression in various human epithelial tumors [65]. Active immunotherapy targeting EGFR aims to elicit potent anti-tumor humoral responses, which offer the advantage of generating a repertoire of specific anti-EGFR antibodies within the body. The DTT-EG3, DTT-EG5, DTT-EG6, and DTT-EG7 vaccines were rationally designed and constructed to induce specific immune responses in mice. Following three consecutive immunizations of mice, high titer levels of anti-EGFR antibodies were detected in mouse serum. Simultaneously, it exhibited a tumor-inhibitory effect in the established A549 mouse tumor model. These discoveries highlight that targeting EGFR is not only promising for passive immunotherapy but also for the active immunotherapy strategy through the construction of vaccines using EGFR epitope peptides.

Immunoinformatics offers a diverse range of epitope prediction and analysis tools to facilitate vaccine development. In order to comprehensively evaluate computer-aided vaccine design, both in vitro and in vivo analyses are indispensable for facilitating the development of effective vaccines. In the preliminary experiments, the constructed DTT-EG3, DTT-EG5, DTT-EG6, and DTT-EG7 vaccines could not only simultaneously trigger specific humoral and cellular responses but also exhibited inhibitory effects on the growth of A549 tumors in both the prevention and treatment models. The accuracy of epitope prediction requires further in-depth verification. The vaccine design based on the antigen epitope prediction method of bioinformatics combined with in vitro and in vivo experiments is a reasonable and convenient approach. The HLA allele frequencies and reference sets for HLA class I [66] and II [67] molecules with the maximum population coverage provided by IEDB can be applied in vaccine development and other areas. With further advancements in immunoinformatics research, supplementation and development thereof can enhance the reliability and accuracy of epitope prediction methods while offering broad application prospects.

Although autoantigens commonly found in cancer patients have been utilized for the development of cancer vaccines, their clinical efficacy has proven to be unsatisfactory [68]. This can be attributed to the inherent low immune response elicited by autoantigens due to immune self-tolerance [69]. The binding of DTT to the EGFR-ECD epitope to construct vaccines, namely, DTT-EG3, DTT-EG5, DTT-EG6, and DTT-EG7, elicited potent antibody responses against DTT and EGFR and stimulated stronger cellular responses compared to the DTT-alone vaccine. When epitopes are grafted onto the C-terminus of DTT via the GS linker, DTT exhibits robust tolerance towards the transplantation of foreign peptides while maintaining their stable conformation. This once again highlights the safety and effectiveness of using DTT as a design carrier for tumor vaccines targeting its own molecules. Simultaneously, DTT can be expressed as a highly soluble protein in *E. coli*, and the engineered DTT-EG recombinant protein also demonstrates soluble expression characteristics in *E. coli*, thereby significantly reducing the production costs of the DTT-EG vaccine. The Th epitope of DTT can be recognized by the human major histocompatibility complex, exhibiting a population coverage exceeding 70% [70]. Due to the extensive genetic polymorphism of human MHC II, the inclusion of universal Th epitopes within DTT is anticipated to encompass a broader population. DT vaccine-derived Th epitopes elicit rapid recall of CD4 memory T cells in previously immunized populations [71]. This DTT-EG vaccine fusion strategy may benefit a large number of cancer patients.

In comparison to monoclonal antibodies or chemotherapy, vaccines necessitate a longer duration for initiating an immune response and exhibit greater reliance on the immunological status. The pre-existing anti-EGFR-specific antibodies elicited by DTT-EG vaccination can effectively control tumor growth and, in conjunction with the immune system, impede and expedite tumor regression. Furthermore, in the therapeutic mouse model, this DTT-EG vaccine strategy exhibited enhanced efficacy against residual and early tumors. According to clinical data, patients with early-stage cancer exhibit a favorable prognosis and prolonged overall survival [72]. The DTT-EG vaccine, which specifically targets EGFR, holds promise for achieving long-term tumor control in these patients following surgical intervention. In comparison to monoclonal antibodies, the DTT-EG vaccine offers a potential solution for alleviating the financial burden on patients. In contrast to peptide cancer vaccines constituted by short peptide sequences of tumor-associated antigens, recombinant protein vaccines composed of DTT carrier proteins and antigenic epitopes possess advantages in terms of chemical stability, breaking self-immune tolerance, and production costs. However, in comparison with short peptide sequences that can be directly bound by MHC I or II on APCs, recombinant proteins involve the processing and presentation of antigens, demanding more in-depth antigen-specific experiments for detection and verification. Relevant studies have indicated that the CIMAvax-EGF therapeutic vaccine, as an EGF-depleting immunotherapy for patients with NSCLC in switch maintenance, is safe and effective [16,17,73,74]. The median survival time (MST) of patients treated with CIMAvax-EGF can reach 14.6 months. The combination of CIMAvax-EGF with TKIs or ICIs might further facilitate the transformation of advanced cancer into a chronic disease [73]. Currently, there is a lack of adequate research and clinical trials to determine whether the DTT + epitope vaccine can be employed as a preventive vaccine for high-risk groups of cancer. Perhaps, it is more applicable in the treatment of related cancers at the early diagnosis stage or in combination with chemotherapy and radiotherapy.

Human EGFR serves as a xenoantigen capable of eliciting specific immune responses in mice and exhibiting cross-reactivity with mouse EGFR, thereby exerting therapeutic effects on EGFR-positive mouse tumors [75]. Previous studies have also demonstrated that prophylactic vaccination with human EGFR peptides can effectively break self-tolerance without inducing systemic autoimmunity, thus impeding tumor development [34]. In our study, the identified epitopes capable of generating anti-EGFR-specific antibodies exhibit a substantial degree of homology with mouse EGFR. Specifically, the homology percentages for EG3, EG5, EG6, and EG7 are 85.71%, 93.33%, 93.33%, and 100.00%, respectively. The cross-reactive responses elicited by DTT-EG3, DTT-EG5, DTT-EG6, and DTT-EG7 vaccines suggest that the high homology of epitope peptide sequences between humans and mice can provide valuable insights for the development of human vaccines. Meanwhile, the high degree of conservation of these sequences also avoids cross-reactivity with other proteins after immunization.

Although we explored and determined the approaches of immune protocols and tumor model construction through rationally designed pre-experiments, in order to further improve the consistency and reliability of the research results and make the results more statistically significant, expanding the sample size in the subsequent research is indispensable. In this experiment, only the ELISA method was employed to detect the release of IFN-γ. If intracellular cytokine staining and the ELISPOT method were added, more in-depth, comprehensive and complete conclusions could be drawn. The DTT-EG3, DTT-EG5, DTT-EG6, and DTT-EG7 vaccines are capable of inducing specific anti-EGFR antibodies. However, whether these antibodies can be involved in the anti-tumor activity of the vaccines requires further investigation. DTT-EG1, DTT-EG2, and DTT-EG4 were excluded due to their failure to effectively induce anti-EGFR-specific antibodies. However, whether they can induce cellular responses requires further experiments for verification in subsequent studies.

The prediction of antigenic epitopes by immunoinformatics tools to assist vaccine development is an effective approach for cost reduction and time-saving. We have demonstrated that DTT is a safe and effective tumor vaccine design carrier for its own molecules, which can enhance the immunogenicity of weak antigen epitopes. The EGFR antigenic epitopes EG3 (HGAVRFSNNPALCNV145-159), EG5 (KDSLSINATNIKHFK346-360), EG6 (VKEITGFLLIQAWPE398-412), and EG7 (LCYANTINWKKLFGT469-483), when combined with DTT to construct recombinant protein vaccines, might constitute a promising strategy for the prevention or treatment of EGFR-positive tumors. Compared with current antigen-based vaccine design methods, the DTT-EG design method is easier and more feasible for clinical cancer immunotherapy. The adopted vaccine design and fusion method may become a feasible approach to developing cancer vaccines.

## Figures and Tables

**Table 1:** Selection of HLA supertypes and alleles in epitope prediction servers.

Servers for Epitope Prediction	HLA Supertype and Allele
NetMHCpan-4.1 and IEDB Consensus-2.18	A01: HLA-A-01:01, HLA-A-26:01, HLA-A-30:02, HLA-A-32:01 A01/A03 (1): HLA-A-30:01 A02: HLA-A-02:01, HLA-A-02:03, HLA-A-02:06, HLA-A-68:02 A03: HLA-A-03:01, HLA-A-11:01, HLA-A-31:01, HLA-A-33:01, HLA-A-68:01 A24: HLA-A-23:01, HLA-A-24:02 B07: HLA-B-07:02, HLA-B-35:01, HLA-B-51:01, HLA-B-53:01 B08: HLA-B-08:01 B44: HLA-B-40:01, HLA-B-44:02, HLA-B-44:03 B58: HLA-B-57:01, HLA-B-58:01 B62: HLA-B-15:01
NetMHCIIpan-3.2 and IEDB Consensus-2.22	Main DR: HLA-DRB1-01:01, HLA-DRB1-07:01, HLA-DRB1-09:01, HLA-DRB1-11:01, DRB1-12:01, HLA-DRB1-15:01, HLA-DRB5-01:01 DR4: HLA-DRB1-04:01, HLA-DRB1-04:05, HLA-DRB1-08:02 DRB3: HLA-DRB1-03:01, HLA-DRB1-13:02, HLA-DRB3-01:01, HLA-DRB1-02:02, HLA-DRB4-01:01

**Table 2:** CTL epitope prediction of EGFR-ECD.

No.	Peptide sequence	Start–End	Supertype and Allele: NetMHCpan-4.1 and IEDB Consensus-2.18
1	YVLIALNTV	88–96	A02 (HLA-A-02:06)
2	MYYENSYAL	111–119	B27 (HLA-B-39:01) A24 (HLA-A-23:01, HLA-A-24:02)
3	YENSYALAV	113–121	B44 (HLA-B-40:01, HLA-B-18:01)
4	AVRFSNNPA	147–155	A01/A03 (HLA-A-30:01)
5	GEFKDSLSI	343–351	B44 (HLA-B-40:02, HLA-B-40:01, HLA-B-44:02)
6	NATNIKHFK	352–360	A03 (HLA-A-68:01)
7	KEITGFLLI	399–407	B44 (HLA-B-44:02, HLA-B-40:02, HLA-B-44:03)
8	YANTINWKK	471–479	A03 (HLA-A-68:01)

**Table 3:** HTL epitope prediction of EGFR-ECD.

No.	Peptide Sequence (HTL Epitopes)	Start–End	Supertype and Allele: NetMHCIIpan-3.2 and IEDB Consensus-2.22	Peptide Sequence (CTL Epitopes)	Sequence Homology (EGFR-Mus Musculus)	Population Coverage: World (MHC Class I and II Combined)
EG1	YVLIALNTVERIPLE	88–102	DRB3 (HLA-DRB1-13:02) DR4 (HLA-DRB1-04:05)	YVLIALNTV	100.00%	11.37%
EG2	RGNMYYENSYALAVL	108–122	Main DR (HLA-DRB1-09:01, HLA-DRB1-07:01, HLA-DRB1-01:01, HLA-DRB1-15:01) DR4 (HLA-DRB1-04:01) DRB3 (HLA-DRB1-13:02)	MYYENSYAL YENSYALAV	73.33%	76.26%
EG3	HGAVRFSNNPALCNV	145–159	DRB3 (HLA-DRB1-13:02) DR4 (HLA-DRB1-04:01)	AVRFSNNPA	85.71%	29.75%
EG4	IGIGEFKDSLSINAT	340–354	DR4 (HLA-DRB1-04:01)	GEFKDSLSI	93.33%	27.46%
EG5	KDSLSINATNIKHFK	346–360	DRB3 (HLA-DRB1-13:02)	NATNIKHFK	93.33%	12.14%
EG6	VKEITGFLLIQAWPE	398–412	DR4 (HLA-DRB1-04:05) Main DR (HLA-DRB1-07:01, HLA-DRB1-01:01)	KEITGFLLI	93.33%	42.76%
EG7	LCYANTINWKKLFGT	469–483	Main DR (HLA-DRB5-01:01)	YANTINWKK	100.00%	5.83% (without HLA-DRB5-01:01)

**Table 4:** Prediction of the recognition of epitope EG1-7 by Balb/c mouse MHC haplotypes.

No.	Peptide Sequence	Mouse MHC-II Haplotypes:IEDB NetMHCIIpan 4.1 BA	CTL Epitopes (9 mer) and Mouse MHC-I Haplotypes and BindLevel: NetMHCpan-4.1 (%Rank)
EG1	YVLIALNTVERIPLE	H2-IAd (low affinity), H2-IEd (low affinity)	IALNTVERI (H-2-Dd, weak binder)
EG2	RGNMYYENSYALAVL	H2-IAd (low affinity), H2-IEd (low affinity)	MYYENSYAL (H-2-Dd, WB/H-2-Kd, SB/H-2-Ld, weak binder) YYENSYALA (H-2-Kd, weak binder)
EG3	HGAVRFSNNPALCNV	H2-IAd (low affinity), H2-IEd (low affinity)	VRFSNNPAL (H-2-Kd, weak binder)
EG4	IGIGEFKDSLSINAT	H2-IAd (low affinity)	None
EG5	KDSLSINATNIKHFK	H2-IAd (low affinity), H2-IEd (low affinity)	INATNIKHF (H-2-Dd, weak binder) SLSINATNI (H-2-Kd, weak binder)
EG6	VKEITGFLLIQAWPE	H2-IAd (low affinity), H2-IEd (low affinity)	TGFLLIQAW (H-2-Dd, strong binder) KEITGFLLI (H-2-Kd, weak binder)
EG7	LCYANTINWKKLFGT	H2-IAd (low affinity), H2-IEd (low affinity)	LCYANTINW (H-2-Dd, weak binder)

## Data Availability

Data will be made available on request.

## Supplementary Materials

The following supporting information can be downloaded at https://www.mdpi.com/article/10.3390/biom14121620/s1, Table S1. Statistical analysis of the specific antibody response induced by DTT-EG in mice (mean ± SD). Table S2. Statistical analysis of the results of cellular responses induced by DTT-EG (mean ± SD). Table S3. Statistical analysis of the experimental outcomes of the mouse tumor model (mean ± SD). Table S4. Statistical analysis of immunohistochemical results in tumor tissues (mean ± SD).
