# Supplementary material for: Rational Design of an Epidermal Growth Factor Receptor Vaccine: Immunogenicity and Antitumor Research
Source: Biomolecules. 2024 Dec 18;14(12):1620. doi: 10.3390/biom14121620 (PMC11726940; doi:10.3390/biom14121620)
Supplement: Supplementary file 1 [file biomolecules-14-01620-s001.zip › biomolecules-3310510-supplementary.pdf]

Supplementary Materials

Table S1. Statistical analysis of the specific antibody response induced by DTT-EG in mice (mean ± SD).

		PBS	DTT	DTT-EG1	DTT-EG2	DTT-EG3	DTT-EG4	DTT-EG5	DTT-EG6	DTT-EG7
Figure 3B (OD450) (two-way ANOVA)	DTT	0.041± 0.002	1.008± 0.122	0.970±0.130	0.758±0.331	1.028±0.054	0.468±0.279	1.200±0.087	1.248±0.021	0.653±0.133
	EGFR	0.042± 0.000	0.043± 0.003	0.214±0.150	0.110±0.079	0.675±0.048	0.089±0.079	0.968±0.159	0.955±0.135	0.690±0.158
Figure 3C (Titer) (one-way ANOVA)		0±0	0±0	125±189	75±50	8000±3200	50±58	8000±3200	9600±3695	11200±3200

Table S2. Statistical analysis of the results of cellular responses induced by DTT-EG (mean ± SD).

		PBS	DTT	DTT-EG3	DTT-EG5	DTT-EG6	DTT-EG7
Figure 4B (SI,%) (one-way ANOVA)		0.98±0.11	1.62±0.20	3.30±0.44	2.91±0.25	2.71±0.42	3.06±0.42
Figure 4C (Lysis,%) (two-way ANOVA)	50:1	21.4±3.6	23.8±4.9	49.6±6.4	49.3±9.4	41.5±4.8	46.8±3.3
	20:1	18.3±2.8	18.1±3.0	32.7±6.2	34.2±2.4	31.0±5.1	32.0±5.9
Figure 4D (pg/mL) (one-way ANOVA)		263.5±59.0	386.3±94.7	729.7±53.3	796.4±98.3	908.4±43.1	763.3±110.5
Figure 4G (%) (two-way ANOVA)	CD4+ T	6.21±0.60	6.03±0.12	11.16±1.86	8.50±0.78	8.50±0.78	9.56±1.82
	CD8+ T	5.68±0.31	6.12±0.26	12.42±1.03	10.36±1.02	9.94±1.08	9.56±0.71

Table S3. Statistical analysis of the experimental outcomes of the mouse tumor model (mean ± SD).

	PBS	DTT	DTT-EG3	DTT-EG5	DTT-EG6	DTT-EG7
Figure 5B (g) (one-way ANOVA)	0.324±0.092	0.292±0.107	0.076±0.053	0.100±0.045	0.104±0.044	0.078±0.043
Figure 5D (g) (one-way ANOVA)	0.418±0.094	0.352±0.065	0.105±0.044	0.107±0.055	0.092±0.058	0.106±0.039

Table S4. Statistical analysis of immunohistochemical results in tumor tissues (mean ± SD).

		PBS	DTT	DTT-EG3	DTT-EG5	DTT-EG6	DTT-EG7
Figure 6C (%) (two-way ANOVA)	CD4+ T	2.67±0.80	2.77±0.65	13.64±3.78	11.35±2.50	12.64±1.74	8.46±1.01
	CD8+ T	2.78±0.42	2.73±0.56	8.99±1.64	9.10±1.56	8.53±1.52	7.01±2.58
